# Insights into the activity of maturation inhibitor PF-46396 on HIV-1 clade C

**DOI:** 10.1038/srep43711

**Published:** 2017-03-02

**Authors:** Dibya Ghimire, Uddhav Timilsina, Tryambak Pratap Srivastava, Ritu Gaur

**Affiliations:** 1 Faculty of Life Sciences and Biotechnology, South Asian University, New Delhi 110021, India

## Abstract

HIV maturation inhibitors are an emerging class of anti-retroviral compounds that inhibit the viral protease-mediated cleavage of the Gag, CA-SP1 (capsid-spacer peptide 1) peptide to mature CA. The first-in-class maturation inhibitor bevirimat (BVM) displayed potent activity against HIV-1 clade B but was ineffective against other HIV-1 clades including clade C. Another pyridone-based maturation inhibitor, PF-46396 displayed potent activity against HIV-1 clade B. In this study, we aimed at determining the activity of PF-46396 against HIV-1 clade C. We employed various biochemical and virological assays to demonstrate that PF-46396 is effective against HIV-1 clade C. We observed a dose dependent accumulation of CA-SP1 intermediate in presence of the compound. We carried out mutagenesis in the CA- SP1 region of HIV-1 clade C Gag and observed that the mutations conferred resistance against the compound. Many mutations inhibited Gag processing thereby reducing virus release in the absence of the compound. However, presence of PF-46396 rescued these defects and enhanced virus release, replication capacity and infectivity of HIV-1 clade C. These results put together identify PF-46396 as a broadly active maturation inhibitor against HIV-1 clade B and C and help in rational designing of novel analogs with reduced toxicity and increased efficacy for its potential use in clinics.

Since the discovery of HIV/AIDS, at least 25 million deaths have been reported and approximately 33 million people are estimated to be infected with HIV-1 ( http://www.who.int/gho/hiv/en/). The management of HIV/AIDS includes using combination of multiple anti-retroviral drugs that act on different viral targets: commonly called HAART (highly active antiretroviral therapy). The U.S. Food and Drug Administration (FDA) has approved more than twenty five different drugs targeting several distinct steps in the viral replication cycle for clinical use^1^ which have increased patient’s life expectancy. Unfortunately, long-term use of these antiretroviral drugs leads to emergence of drug-resistant viruses^2^,^3^. Hence, it is extremely important to continuously identify and develop new compounds as potent antivirals against HIV-1.

During or shortly after HIV-1 release from the infected cell, the viral protease (PR) – cleaves polyprotein Gag precursor (Pr55^Gag^) to individual proteins: MA (matrix), CA (capsid), NC (nucleocapsid) and p6. This step in HIV-1 life cycle termed as viral maturation is one of the essential steps required to produce mature and infectious virions^4^. The proteolytic processing of Gag occurs in a highly ordered fashion to release the mature proteins from the two spacer peptides SP1 and SP2^4^,^5^,^6^. The rate of cleavage differs at each step with the last step involving the release of SP1 from the C-terminus of CA (CA-CTD) being the rate limiting step^6^,^7^,^8^. These mature CA monomers arrange in a closed hexagonal lattice consisting of 12 CA pentamers which form a conical shell surrounding the viral genome which exhibits fullerene- like geometry^9^,^10^,^11^. Unlike the mature core, the immature HIV-1 Gag shell forms a sphere that is interrupted by large discontinuities^12^. It was recently reported that the immature HIV-1 CA-CTD-SP1 Gag fragment assembles to form a hexamer resembling a goblet in which the main CA-CTD folds to form the cup and contacts the tightly packed 6-helix bundle formed by CA-CTD-SP1junction helices in the stem. The CA-SP1 cleavage site is buried inside the helical barrel and is inaccessible to PR unless the 6-helix bundle unfolds^13^,^14^.

Since virus maturation is critical for the production of infectious virions, maturation inhibitors (MI) have evolved as an emerging class of anti-HIV-1 compounds. To date, two different classes of MI have been identified- (I) betulinic acid derivatives^15^,^16^ and (II) a pyridone-based compound PF-46396 ({1-[2- (4-tert-butylphenyl)-2-(2,3-dihydro-1H-inden-2-ylamion)ethyl]-3- (trifluromethyl)pyridin-2(1H)-one})^17^. Bevirimat (BVM), a class I betulinic acid derivative also known as DSB (3-*0-(3*′*-3*′-dimethylsuccinyl) betulinic acid) or PA-457 is the first-in-class MI. BVM disrupts the cleavage of CA from SP1 leading to the accumulation of CA-SP1 intermediate^18^,^19^,^20^. This inhibitory activity of BVM is partial as even at high concentrations of the compound, mature CA is still observed^21^. Passaging virus in the presence of BVM in cell culture resulted in the production of resistant virus, which had acquired mutations near the CA-SP1 cleavage site^18^,^19^,^22^,^23^,^24^. BVM failed in phase II clinical trials as it displayed efficacy in only half of the HIV patients^25^. The failure of the compound was attributed to the presence of polymorphisms in the SP1 region, specifically SP1-V7A which reduced the sensitivity of HIV-1 to BVM^26^,^27^,^28^,^29^,^30^. This led to termination of clinical trials in 2010. Recently, BVM analogs have been identified that displayed potent activity against multiple HIV-1 clades^31^,^32^,^33^,^34^,^35^,^36^. Apart from BVM, two-second generation MIs, GSK 2828232 and BMS-955176 also demonstrated promising antiviral activity against HIV-1 isolates containing polymorphic SP1 region and are currently in phase I and phase II clinical trials respectively^15^.

The second class of MI is the pyridone-based compound PF-46396. In 2009, Blair and colleagues reported that this compound inhibited CA-SP1 processing in a manner similar to BVM^17^. Although PF-46396 was found to be less potent than BVM, its activity was unaffected by polymorphisms in the SP1 region (particularly V7A) unlike BVM^21^. Furthermore, virus selection experiments using HIV-1 clade B molecular clone, NL4–3, led to identification of mutations in three distinct domains in Gag: CA-SP1 boundary region, CA-CTD residue 201, and CA major homology region (MHR). These results suggested that both PF-46396 and BVM bind to similar regions in HIV-1 Gag. Furthermore, addition of PF-46396 antagonized the activity of BVM suggesting that the two compounds share a portion of a putative binding pocket involving the CA: MHR and SP1 regions of HIV-1 Gag^17^,^21^. Interestingly, mutations in the MHR region conferred PF-46396 compound-dependence on the virus^21^. Recently, Murgatroyd *et al*. used a series of PF-46396 analogues to dissect the role of its functional groups and reported that the *tert*-butyl group in the PF-46396 phenyl ring is essential for its antiviral activity and Gag binding^37^.

MIs stabilize the immature HIV-1 Gag lattice^38^. Gag polymorphisms and mutations that confer resistance to BVM and PF-46396 allow the proteolysis at CA-SP1^20^,^22^,^39^. Furthermore, in case of MI-dependent mutations, the Gag hexamer is destabilized and inhibits virus assembly in the absence of the compound^21^. Mutations like T8I stabilize the immature Gag lattice such that the proteolysis is inhibited even in the absence of the MIs^40^. Mapping of the BVM and PF-46396-resistant mutations onto the recently solved CA-CTD-SP1 structure also showed that the positions of these resistant mutations are predominately located at the protein-protein interfaces within the CA-SP1 lattice and not at the putative compound binding site at the CA-SP1 junction helix^14^. Collectively, these pieces of information support that MIs stabilize the 6-helix bundle and prevent its unfolding^13^,^14^.

The activity of PF-46396 has been tested extensively on HIV-1 clade B^17^,^21^,^37^. However, nearly 50% of HIV infections worldwide are caused by HIV-1 clade C, which is predominant mainly in South Asian countries such as India, China and Eastern and Southern Africa^41^. In 2009, Blair *et al*. had reported poor antiviral activity of PF-46396 against HIV-1 clade C isolate (98IN022)^17^. The two HIV-1 clades (B and C) differ in terms of sequences, structure, and antigenic variance, which not only affects the biological properties of virus and immune responses towards it, but also the susceptibility to existing potential anti-retroviral drugs as well as evolution of drug resistance^36^. Hence, for successful development of PF-46396 as potential MI, it is imperative to test its activity against other HIV-1 clades, in particular HIV-1 clade C in more detail. In this study, we tested the activity of PF-46396 against HIV-1 clade C using various biochemical and virological assays and observed that the compound displayed strong activity against clade C. *In vitro* mutagenesis studies in the CA-CTD-SP1 region of HIV-1 clade C identified amino acid residues, which inhibited Gag processing and conferred resistance and compound-dependence to the compound. Our study highlights that PF-46396 has the potential to be developed as broadly active MI, which is essential for its success in clinics.

## Results

### Effect of PF-46396 on CA-SP1 processing and replication

**Figure 1:**
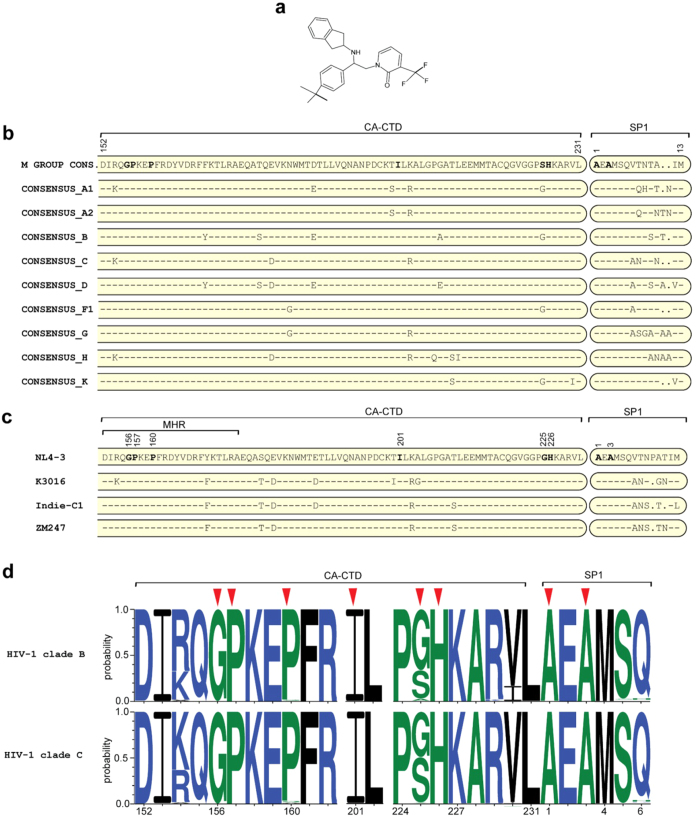
Maturation inhibitor PF-46396 and natural polymorphisms at HIV-1 CA C-terminal and SP1 domains. (**a**) Chemical structure of the compound PF-46396 ({1-[2- (4-tert-butylphenyl)-2-(2,3-dihydro-1H-inden-2-ylamion)ethyl]-3- (trifluromethyl)pyridin-2(1 H)-one}). (**b**) Amino acid sequence alignment of the CA-CTD (amino acids numbers 152–231 of CA) and SP1 region of HIV-1 group M viruses. (**c**) Amino acid sequence alignment of the CA-CTD (amino acids numbers 152–231 of CA) and SP1 region for HIV-1 clade B clone NL4-3 and three HIV-1 clade C clones (K3016, IndieC1 and ZM247) used in this study. Amino acid residues comprising major homology region (MHR) are indicated. Positions of amino acid residues that confer resistance to PF-46396 in HIV-1 clade B are shown in bold and numbered. Hyphens indicate amino acids identical to the NL4-3 sequence (shown at the top); dots indicate gaps. (**d**) Sequence logos for HIV-1 clade B (3050 sequences) and clade C (1778 sequences) CA-CTD-SP1 residues derived from the curated Los Alamos HIV-1 sequence database. Positions of amino acid residues are numbered (X-axis). Conservation of a residue is shown as a function of probability of its occurrence at a particular position (Y-axis). Red arrows indicate positions of amino acids selected for mutagenesis in this study. Blue, green and black indicates hydrophilic, neutral and hydrophobic amino acids respectively.

**Figure 2:**
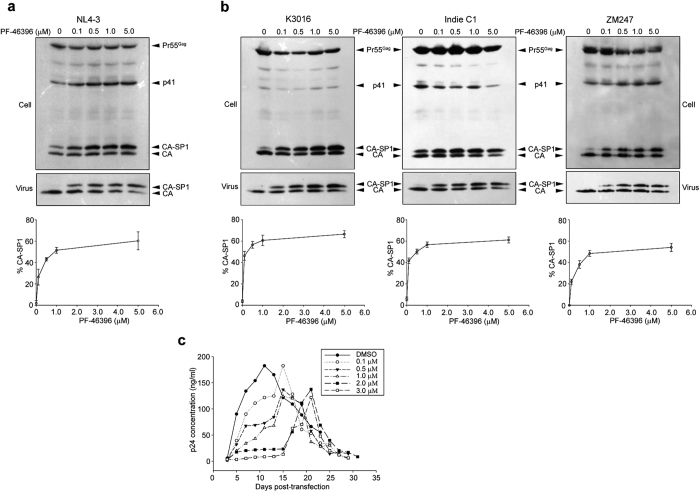
PF-46396 is active against HIV-1 clade C. CA-SP1 accumulation assay of (**a**) HIV-1 clade B clone NL4-3 and (**b**) HIV-1 clade C clones in the presence of PF-46396. HEK-293T cells were transfected with (**a**) HIV-1 clade B clone NL4-3 or (**b**) HIV-1 clade C clones K3016, IndieC1 and ZM247. Cells were treated with increasing concentrations of PF-46396 (0.1–5.0 μM) or with DMSO only. Cell- and virion-associated Gag proteins were detected by western blotting. Positions of Pr55^Gag^, p41, CA, and CA-SP1 are indicated. Gel images shown here are representative of three independent experiments. Quantification of virion-associated % CA-SP1 relative to total CA + CA-SP1 is presented in the graphs. Error bars indicate standard deviations. (**c**) Replication kinetics of HIV-1 clade C clone K3016 in the presence or absence of PF-46396. HUT-R5 T-cells were transfected with K3016 WT and propagated in the presence of 0–3.0 μM of PF-46396. Virus replication was monitored by quantifying HIV-1 p24 concentration in the culture supernatants. This is a representative of three independent experiments.

Most of the previous studies on antiviral activity of PF-46396 (depicted in Fig. 1a) have been carried out against HIV-1 clade B virus^17^,^21^,^37^. We aligned the CA-CTD-SP1 sequences of HIV-1 M group isolates from Los Alamos HIV-1 sequence database ( http://www.hiv.lanl.gov/content/index) and observed subtle differences in terms of sequences in the CTD of CA and specifically in the SP1 region (Fig. 1b), which forms the putative binding pocket of the compound. It has been previously reported that BVM is inactive against HIV-1 clade C due to these inherent differences in its sequences in the CA-SP1 region, specifically the QVT motif in the SP1 region^30^,^36^. Whether PF-46396 also displayed similar behavior against HIV-1 clade C remained unknown. We aimed at elucidating the efficacy of PF-46396 in inhibiting the CA-SP1 processing of HIV-1 clade C virus. For our experiments we used three HIV-1 clade C molecular clones isolated from different geographical locations: ZM247 and K3016 (isolated from South Africa) and Indie-C1 (isolated from India). HIV-1 clade B molecular clone, NL4-3 was used as control. Amino acids sequence variations in the CA-CTD-SP1 Gag region of these four viruses are shown in Fig. 1c. HEK-293T cells were transfected with HIV-1 clade B or C DNA in the absence or presence of increasing concentrations of PF-46396 (0.1–5.0 μM). Cell lysates and pelleted viral supernatants were separated on SDS-PAGE followed by western blotting using HIV-IgG antibody. As can be seen in Fig. 2a, a dose-dependent accumulation of cell- and virion-associated CA-SP1 was observed with increasing concentration of PF-46396 using NL4-3 as reported previously^21^. Interestingly, the three HIV-1 clade C clones also demonstrated a similar dose-dependent kinetics with 5.0 μM of PF-46396 displaying nearly 60% accumulation of CA-SP1 intermediate (Fig. 2b). These results suggested that unlike BVM, PF-46396 was broadly active against the two most infectious HIV-1 clades B and C.

Next we wanted to test whether PF-46396 could inhibit the replication of HIV-1 clade C in cell culture. Since all three HIV-1 clade C clones tested behaved similarly in previous experiments, we decided to perform the subsequent experiments using K3016. The *de novo* selection experiments were performed using HUT-R5 T-cells, which carry CCR5 co-receptor. Briefly the cells were transfected with K3016 DNA using DEAE-dextran and maintained in the presence of 0–3.0 μM of PF-46396. The replication of virus was monitored by quantitating HIV-1 p24 antigen released in the culture supernatant (Fig. 2c). In the absence of compound, the wild type (WT) K3016 virus replication peaked approximately eleven days post-transfection. With increasing concentration of the compound, we observed a delay in virus replication. In the presence of 0.1, 0.5 and 1.0 μM of the compound, a delay of only 4 days was observed with the virus replication peaking at day 15 in all cases. However, in the presence of 2.0 and 3.0 μM of PF-46396, we observed a significant delay of almost 10–12 days as compared to virus without compound (Fig. 2c). To determine whether the virus has acquired any mutations, the infected cells were collected on the days of peak p24 production and genomic DNA was isolated. The entire Gag coding region of the provirus was PCR amplified, purified and sequenced as described in Methods. We did not observe any changes in the entire Gag region of the revertant virus as compared to control. These results suggested that concentration of the compound (2.0–3.0 μM) might not be sufficient to confer selection pressure on the virus although the compound effectively inhibited the CA-SP1 cleavage at that concentration (Fig. 2b). We next repeated the experiment with increasing concentration of the compound to 4.0 and 5.0 μM. Unfortunately, the compound was cytotoxic to HUT-R5 and PM1 T-cells at that concentration and led to cell death as evident from the cytotoxicity assay (data not shown). To summarize, PF-46396 delayed HIV-1 clade C virus replication *ex vivo*.

### Construction and characterization of PF-46396-resistant HIV-1 clade C mutants

Waki *et al*. had previously identified the putative residues in HIV-1 clade B Gag, which might be involved in binding PF-46396^21^. Since, the compound displayed strong activity against both the HIV-1 clades B and C, we presumed that the contact residues on HIV-1 clade C Gag might be similar to clade B. To verify this, we introduced all the mutations (previously selected in NL4-3)^21^ in the backbone of K3016 DNA. The mutations created are depicted in Table 1. None of the mutations were inherently present in K3016, ZM247 or IndieC1as evident from the sequence alignment shown in Fig. 1c. Analysis of CA-CTD-SP1 sequences derived from the curated Los Alamos HIV-1 sequence database showed that these residues are highly conserved across HIV-1 clades except at CA-CTD 225 position (Table 1, Fig. 1d).

**Figure 3:**
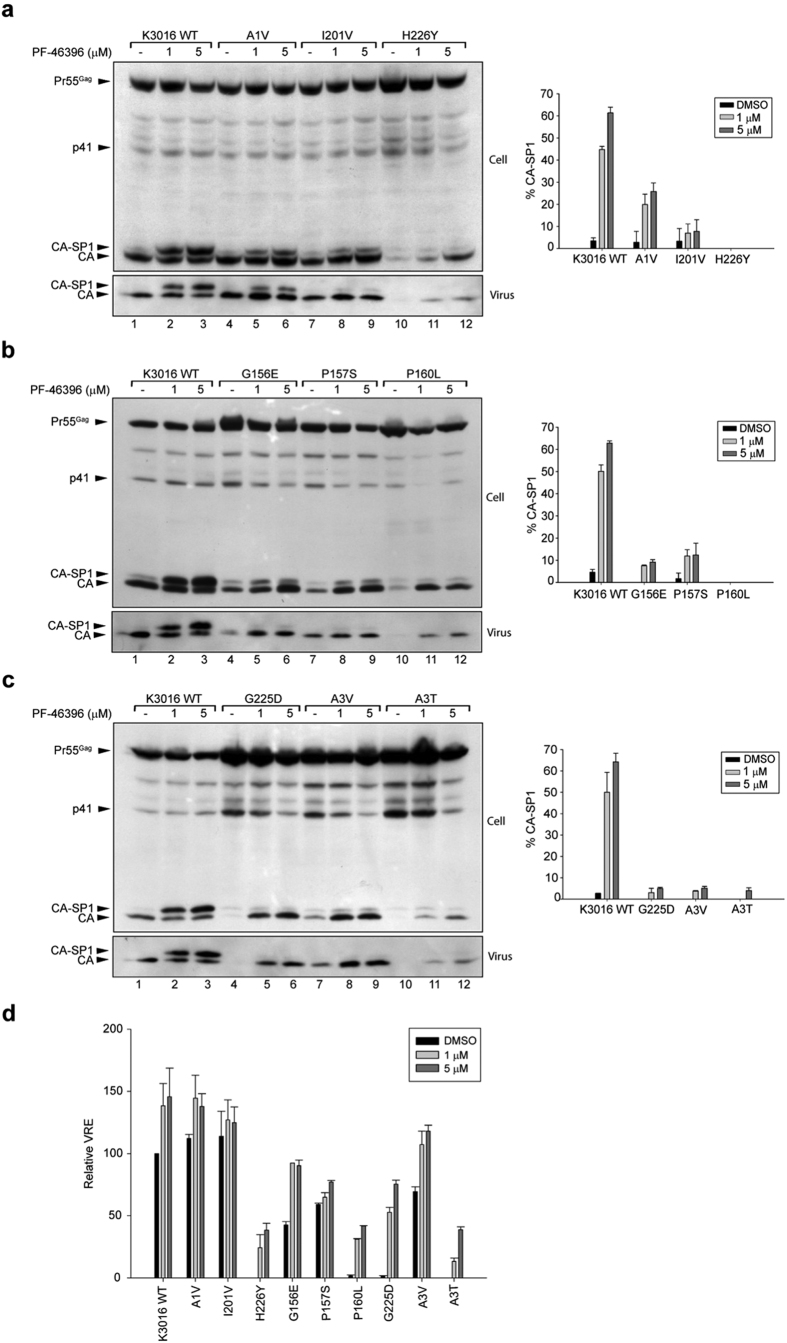
Characterization of PF-46396-resistant mutants. CA-SP1 accumulation assay of (**a**) HIV-1 clade C K3016 WT, K3016 A1V, I201V and H226Y mutants (**b**) HIV-1 clade C K3016 WT, K3016 G156E, P157S and P160L mutants (**c**) HIV-1 clade C K3016 WT, K3016 G225D, A3V and A3T mutants. HEK-293T cells were transfected with HIV-1 clade C clone K3016 WT and indicated K3016 mutants. Cells were treated with PF-46396 (1.0 & 5.0 μM) or with DMSO only. Cell- and virion-associated Gag proteins were detected by western blotting. Positions of Pr55^Gag^, p41, CA, and CA-SP1 are indicated. Lanes are numbered at the bottom of the gel. Gel images shown here are representative of three independent experiments. Quantification of virion-associated % CA-SP1 relative to total CA + CA-SP1 is presented in the graphs on the right side of each panel. (**d**) Relative virus release efficiency (VRE) of PF-46396-resistant mutants in the absence and presence of the compound. VRE is calculated as % of [virus-associated CA + CA-SP1]/[total (cell + virus-associated) Gag] relative to K3016 WT from (**a**–**c**) gel images. Error bars indicate standard deviations from three independent experiments.

To examine whether the mutations conferred resistance to K3016 against PF-46396, we first determined their role in inhibiting CA-SP1 processing. HEK-293T cells were transfected with HIV-1 clade C mutant molecular clones in the absence or presence of 1.0 and 5.0 μM of PF-46396. Cell lysates and pelleted viral supernatants were separated on SDS-PAGE followed by western blotting as described before. As can be seen in Fig. 3a, mutation in the SP1 region (A1V) conferred partial resistance against the compound as evident from reduction in CA-SP1 accumulation to ~15% and 25% at 1.0 and 5.0 μM compound as compared to the wild type K3016 which showed ~45% and 62% at the same concentration (compare lanes 5–6 with 2–3). Whereas, the CA mutant I201V conferred nearly complete resistance against the compound (less than 8% CA-SP1 accumulation at 5.0 μM concentration) as evident from reduction in CA-SP1 accumulation (Fig. 3a, compare lanes 8–9 with 2–3). These results were similar to NL4–3^21^. Furthermore, HIV-1 clade C CA mutant, H226Y was also found to be resistant and compound dependent. Unlike NL4-3, a severe reduction in both cell- and virus-associated CA was observed suggesting a block in virus maturation and release (Fig. 3a, lane 10). The defect in Gag processing was improved by addition of the compound, thus enabling partial rescue in virus release (Fig. 3a, lanes 11–12). The virus release efficiency of H226Y increased to ~40% in the presence of 5.0 μM of the compound as compared to the K3016 WT (Fig. 3d).

We further analyzed the remaining mutants and observed that the CA MHR mutants: G156E, P157S and P160L and mutation in the CTD of CA (G225D) and SP1 region (A3V and A3T) conferred resistance to PF-46396 as evident from the reduction in CA-SP1 accumulation (Fig. 3b,c). It is significant to note that the clade C mutants P160L, G225D and A3T showed severe defect in virus release like clade B. In contrast two of the clade C mutants G156E and P157S showed a substantial amount of virus release in the absence of compound (Fig. 3d), unlike clade B^21^. The virus release efficiency of all the mutants was improved in the presence of PF-46396 suggesting compound-dependence (Fig. 3d). These results indicated that the clade B-resistant mutations also conferred resistance to clade C Gag albeit the effect of these mutations on Gag processing varied slightly between the two clades.

### Replication profile of PF-46396-resistant mutants

**Figure 4:**
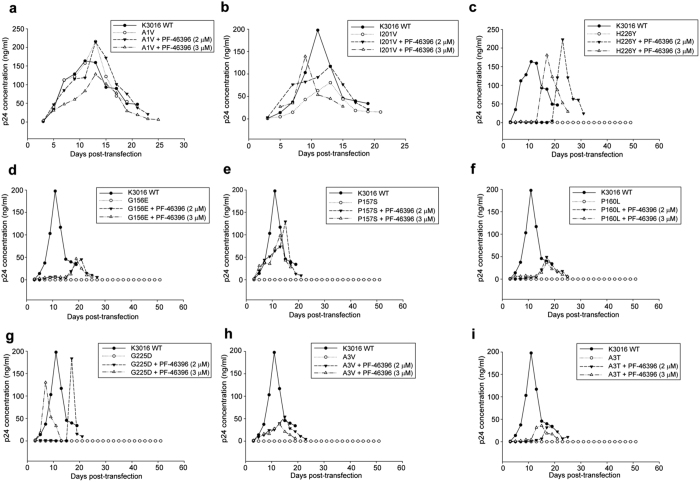
Replication kinetics of PF-46396-resistant mutants. The HUT-R5 T-cells were transfected with K3016 mutants (**a**) A1V, (**b**) I201V, (**c**) H226Y, (**d**) G156E, (**e**) P157S, (**f**) P160L, (**g**) G225D, (**h**) A3V and (**i**) A3T. K3016 WT was also included as an experimental control. Transfected cells were propagated in the presence of 2.0 & 3.0 μM of PF-46396 or with DMSO only. Virus replication was monitored by quantifying HIV-1 p24 concentration in the culture supernatant. This is a representative of two independent experiments.

We next wanted to determine the replication capacity of the PF-46396 mutants in the presence or absence of compound. HUT-R5 T-cells were transfected with K3016 WT and mutant viral DNA and maintained in the presence of 0–3.0 μM of PF-46396. Virus replication kinetics was monitored by quantitating HIV-1 p24 antigen in culture supernatant. The replication capacity of virus with SP1 mutation (A1V) was similar to wild type virus even in the presence of compound suggesting that the mutation conferred resistance to the virus (Fig. 4a). The CA mutant I201V replicated faster in the presence of 3.0 μM of compound confirming partial compound dependence (Fig. 4b), though we did not see significant differences in virus release efficiency with increasing concentrations of PF-46396 (Fig. 3d). All the remaining viruses with mutation that conferred resistance and compound-dependence could replicate only in the presence of compound (Fig. 4c–i). None of these viruses could replicate in the absence of compound as monitored till more than 50 days. In case of clade C mutants H226Y, P160L, G225D and A3T, we had observed negligible virus released in the absence of compound, which could explain their failure to replicate. However, it is interesting to note that the clade C mutants G156E, P157S and A3V showed significant virus released in the absence of compound (Fig. 3d), but still failed to replicate suggesting that the virus particles formed might be defective or non-infectious. These results slightly differed from clade B in which the mutants P157S and H226Y in addition to A1V and I201V could replicate in the absence of compound also.

### Infectivity of PF-46396-dependent and resistant mutants in the presence of compound

**Figure 5:**
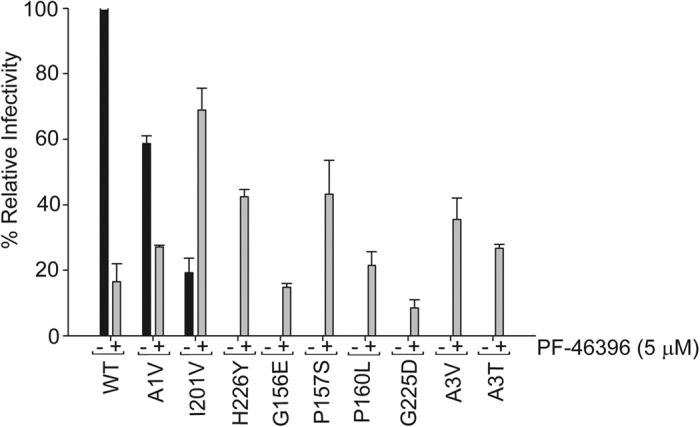
Infectivity of PF-46396-resistant mutants. TZM-bl cells were infected with p24 normalized K3016 WT or mutant virus that has been either PF-46396 (5.0 μM) treated or DMSO-treated. The cells were lysed after 48 h post infection and assayed for luciferase activity. Quantitative data for levels of infectivity relative to DMSO control-treated sample are shown (n = 3). Error bars indicate standard deviations.

It is well known that defects in Gag processing results in release of immature and non-infectious viruses. Hence, we wanted to confirm if the virus released after transfection of HIV-1 clade C mutants was non-infectious. To measure infectivity, we performed the TZM-bl cell-based single-round infectivity assay. The wild type and mutant viruses were produced in the absence (DMSO only) or presence of 5.0 μM of compound in HEK-293T cells. These viruses were used to infect TZM-bl cells for 48 hrs. The infectivity of viruses was measured as a function of Tat-induced luciferase (Luc) reporter gene expression 48 h post-infection. As can be seen in Fig. 5, presence of compound PF-46396 reduced infectivity of K3016 WT and A1V mutant only. All the remaining mutants produced virus that was non-infectious in the absence of compound and displayed an increase in infectivity in the presence of PF-46396. This observation could explain the incapacity of G156E, P157S and A3V to replicate despite significant virus released. The CA mutant I201V virus showed approximately three-fold increase in infectivity in the presence of 5.0 μM compound compared to its DMSO control (Fig. 5). These results suggested that the presence of compound rescued defects in Gag processing thereby increasing virus release and infectivity.

## Discussion

In this study, we evaluated the activity of maturation inhibitor PF-46396 against HIV-1 clade C. Previous studies using this compound had focused primarily on HIV-1 clade B molecular clone NL4-3 (WT and V7A mutant)^17^,^21^,^37^. We selected three well characterized clade C molecular clones K3016, ZM247 and Indie-C1 for our experiments and demonstrated that PF-46396 strongly inhibited the PR-mediated CA-SP1 cleavage in all the cases. The compound delayed viral replication at low concentrations but was toxic to PM1 and HUT-R5 T-cells at higher concentrations. Due to this reason we were unable to select for mutations conferring resistance to the compound in HIV-1 clade C. Previous studies have also reported increased cytotoxicity of this compound to cell lines^17^.

Waki *et al*. had identified residues involved in conferring resistance to the compound in HIV-1 clade B^21^. We examined whether the same residues also imparted resistance to HIV-1 clade C. These residues lie in the three different pockets in the HIV-1 Gag: MHR, CA-CTD and SP1. Changing alanine to valine (A1V) at the first amino acid residue in the SP1 region conferred resistance to PF-46396 in HIV-1 clade C. This residue lies at the junction of CA-CTD and SP1 hence substitution of SP1 A1 residue to V may destabilize the CA: SP1 junction helix (comprising of CA-CTD: 224-231 and SP1: 1–8 residues) in the immature virions thereby allowing the protease to cleave the CA-SP1 junction. Another CA mutation I201V conferred resistance to HIV-1 clade C as evident from the lack of CA-SP1 accumulation in the biochemical assays. Surprisingly, this mutant virus replicated faster and peaked earlier than the wild type in our replication experiments, in the presence of 3.0 μM of PF-46396, indicating compound dependence. This compound dependence of the I201V mutant was not observed in HIV-1 clade B. The infectivity of I201V mutant virus was also increased three fold in the presence of high concentrations of PF-46396 as compared to control (Fig. 5). Though CA-CTD 201 residue lies far away from the CA: SP1 junction in the primary structure of CA, structural analysis of immature HIV-1 Gag CA-CTD-SP1 lattice showed that helix 9/10 loop comprising CA-CTD 201 residue is in close proximity to junction helix and may be involved in the formation of CA hexagonal lattice^14^. Additional difference between the two HIV-1 clades was observed in case of mutation in another CA-CTD residue histidine to tyrosine at position 226 (H226Y). The clade C H226 mutant virus release, replication capacity and infectivity were inhibited in the absence of compound in contrast to NL4-3^21^. Addition of compound rescued all these defects (Figs 3a, 4c and 5). Since CA-CTD residue 226 lies in the CA: SP1 junction helix; we speculated that mutation at this position may impact virus assembly and release. Indeed mutation to alanine at this position (H226A) abolished virus assembly as reported previously^13^. HIV-1 clade C MHR mutants (G156E, P157S and P160L), CA mutant G225D and SP1 mutants (A3V and A3T) were observed to be resistant and compound-dependent as evident from CA-SP1 accumulation assay similar to clade B^21^,^37^. However, the replication capacity of P157S was inhibited in clade C in contrast to clade B in the absence of compound suggesting more severe defects in virus maturation induced by this mutation in clade C.

Though, neither the CA-CTD-SP1 6-helix bundle nor the SP1 region is a universal entity of retroviral Gag shell^42^,^43^, mutagenesis studies highlighted that virtually the entire CA-SP1 junction is required for the assembly of the immature HIV-1 Gag^13^. The maturation switch region comprising a type II β- turn (CA-CTD residues 220–224; GVGGP motif) and an α-helix spanning the CA-SP1 junction (CA-CTD residues 225–231; SP1 residues 1–6) formed the HIV-1 Gag assembly determinant and mutations in this region mostly abolished virus assembly^13^,^14^. Resistant mutations identified in HIV-1 clade B conferred resistance to clade C Gag as well. Most of the mutants in HIV-1 clade C except SP1 A1V were found to exhibit compound dependence. Binding of PF-46396 to these mutant viruses corrected impaired folding of Gag in these regions and enhanced virus release. A comparative analysis of the structure of CA-SP1 region of HIV-1 clade B and C Gag would be helpful in analyzing the interactions of the compound and could answer the differences in phenotype of some of the mutants. The present study has highlighted that PF-46396 is effective against HIV-1 clade C which is responsible for nearly 50% of HIV infections worldwide. These results could serve as a useful platform for rational designing of PF-46396 analogs with low toxicity and broad specificity for their success in clinics.

## Methods

### Compound, plasmids, tissue culture and transfections

The compound PF-46396 was obtained from Pfizer, USA under their compound transfer program (CTP). The lyophilized compound was dissolved in dimethyl sulfoxide (DMSO) to generate 500 μM stock solutions and stored in the dark at −80 °C. HIV-1 clade B clone pNL4-3 (a kind gift from Dr Eric O. Freed, National Cancer Institute, NIH, USA), HIV-1 clade C clones; K3016 (a kind gift from Dr. Christina Ochsenbauer, University of Alabama, USA; GeneBank accession: KC156129), ZM247 (a kind gift from Dr Eric O. Freed, National Cancer Institute, NIH, USA; GeneBank accession: FJ496200.1) and pIndie-C1 (a kind gift from Dr. Uday Ranga, JNCASR, India; GeneBank accession: AB023804) were used in this study. Plasmid DNAs were purified using Thermo Fisher Scientific Plasmid Maxiprep kit as per manufacturer’s instructions. HEK-293T and TZM-bl cells were maintained in Dulbecco’s modified Eagle’s medium (DMEM) containing 10% fetal bovine serum (FBS). PM1 and HUT-R5 T-cells were maintained in RPMI-1640 medium supplemented with 10% FBS. HEK-293T and HUT-R5 cells were transfected with Lipofectamine 2000 (Invitrogen, USA) and DEAE dextran, respectively as described previously^36^,^44^.

### Site-directed mutagenesis

All mutagenesis was performed on the K3016 WT plasmid DNA backbone with synthetic complementary oligonucleotides (IDT, Belgium) using the QuickChange II XL site-directed mutagenesis kit (Agilent Technologies, USA) as per manufacturer’s instructions. Synthetic complementary oligonucleotides used were: G156E; 5′-AGCATTTTGGACATAAAACAAGAGCCAAAGGAACCCTTCAG-3′, P157S; 5′-TTGGACATAAAACAAGGGTCAAAGGAACCCTTCAGAG-3′, P160L; 5′-AAACAAGGGCCAAAGGAACTCTTCAGAGATTATGTAGAC-3′, I201V; 5′-GCGAACCCAGATTGTAAGATCGTTTTAAGAGGATTAGGACC-3′, G225D; 5′-GTGGGAGGACCCGACCACAAGGCAAGG-3′, H226Y; 5′-GGAGGACCCGGCTACAAGGCAAGGG-3′, A1V; 5′-GGCAAGGGTGTTGGTTGAGGCAATGAGCC-3′, A3V; 5′-AGGGTGTTGGCTGAGGTAATGAGCCAAGCAAAC-3′, and A3T; 5′-CAAGGGTGTTGGCTGAGACAATGAGCCAAGCAAAC-3′. The change from WT DNA sequence is underlined. Mutations were confirmed by DNA sequencing using primers K3016-1306F (5′-ATCAGAAGGAGCCACTCCAC-3′) and K3016-1807R (5′-TGTAGCCCCTGGTCCTAATC-3′).

### Virus replication assays

For multi-cycle replication assay, 5 × 10^6^ HUT-R5 cells were transfected with 5 μg HIV-1 clade C (K3016) DNAs and maintained in the presence of 0–3.0 μM of PF-46396. Cells were split in the ratio 2:1 on every 3^rd^ day. Virus replication kinetics was monitored by quantitating p24 antigen using HIV-1 p24 Antigen Capture kit (ABL, USA). To identify mutations that conferred resistance to the compound, the cell pellets were collected on the days of peak HIV-1 p24 concentrations and genomic DNAs were extracted using Blood DNA extraction kit (Qiagen, Germany). The entire Gag coding region of the provirus was PCR amplified using primers K3016-752F (5′-CCGAATTTTATTTGACTAGCGGAG-3′) and K3016-2307R (5′-CTGGCCCCCTACTTTTATTGTG-3′). The 1.5 kb PCR products were purified with ExoSAP-IT PCR product cleanup reagent (Affymetrix, USA) and sent for sequencing using primers K3016-752F (5′-CCGAATTTTATTTGACTAGCGGAG-3′), K3016-1306F (5′-ATCAGAAGGAGCCACTCCAC-3′), K3016-1795F (5′-ACCAGGGGCTACATTAGAAG-3′), K3016-1319R (5′-TGGCTCCTTCTGATAATGCTG-3′), K3016-1807R (5′-TGTAGCCCCTGGTCCTAATC-3′), and K3016-2307R (5′-CTGGCCCCCTACTTTTATTGTG-3′).

### CA-SP1 accumulation assay

HEK-293T cells grown in six well plates to about 80% confluency were transfected with HIV-1 DNAs (3 μg). The culture medium was replaced with fresh DMEM after 24 h post-transfection and incubated for another 2 h. PF-46396 was maintained in the culture throughout transfection. Cellular debris was removed from the culture supernatant by centrifugation at 845 × *g* for 3 min. The clarified supernatants were filtered using 0.45 μm pore size filter disc to remove residual cellular contaminants. The virus was pelleted by ultra centrifugation at 210,100 × *g* for 1 h at 4 °C using SW41Ti rotor (Beckman Coulter, USA). The virus pellet was resuspended in radioimmunoprecipitation assay (RIPA) buffer (50 mM Tris-HCl pH 8.0, 150 mM sodium chloride, 1.0% NP-40, 0.5% sodium deoxycholate, 0.1% SDS) containing 1 × protease inhibitor cocktail (Roche, Germany). Transfected cells were washed and collected in 1 × phosphate buffered saline (PBS). The cell pellets obtained after centrifugation at 845 × *g* for 5 min were lyzed in 1 × RIPA buffer. To measure accumulation of CA-SP1, immunoblot analysis of cell- and virus-associated proteins was performed. The cell and viral lysates were subjected to SDS-polyacrylamide gel electrophoresis (15% gel); proteins were transferred to polyvinylidene difluoride membranes and reacted with HIV-IgG (NIH AIDS Reagent Program; catalog no. 3957) followed by incubation with HRP-conjugated anti-human secondary antibodies (GE Healthcare, UK). The proteins were visualized by enhanced chemiluminescence (Pierce, USA) and the bands were quantified using ImageJ software ( http://imagej.nih.gov/ij/).

### Viral infectivity assay

Virus stocks were prepared by transfecting HEK-293T cells with HIV-1 DNAs (3 μg). Cells were maintained for 24 h post-transfection with or without 5μM PF-46396, centrifuged and filtered to remove residual cell debris. The virus stocks were quantified for p24 antigen using HIV-1 p24 Antigen Capture kit (ABL, USA). Equal volumes (300 μl) of HIV-1 p24 normalized virus supernatants (5 ng HIV-1 p24) were used to infect TZM-bl cells (5 × 10^4^/well) in the presence of 20 μg DEAE-dextran per ml in 24 well plate. Single-round infectivity assays were performed as previously described^45^. The luciferase activity in the cell lysates was measured using the Steady-Glo luciferase assay kit (Promega, USA) following manufacturer’s recommendations.

## Additional Information

**How to cite this article**: Ghimire, D. *et al*. Insights into the activity of maturation inhibitor PF-46396 on HIV-1 clade C. *Sci. Rep.* **7**, 43711; doi: 10.1038/srep43711 (2017).

**Publisher's note:** Springer Nature remains neutral with regard to jurisdictional claims in published maps and institutional affiliations.

## Figures and Tables

**Table 1:** PF-46396-resistant mutations generated in this study and their conservation status among HIV-1 clades.

CA-CTD mutations	Conservation of residues (% frequency)#	SP1 mutations	Conservation of residues (% frequency)#
G156E	99.75	A1V*	99.72
P157S	99.97	A3V	99.89
P160L	99.97	A3T	99.89
I201V	99.66		
G225D	49.30		
H226Y	99.86		

^*^SP1: A1V mutation was not selected in the presence of PF-46396.

^#^Amino acid percentage frequency by position derived from the curated Los Alamos HIV-1 sequence database (3556 sequences of HIV-1 Major subtypes only).
